# Multiscale Approximate Entropy for Gait Analysis in Patients with Neurodegenerative Diseases

**DOI:** 10.3390/e21100934

**Published:** 2019-09-25

**Authors:** An-Bang Liu, Che-Wei Lin

**Affiliations:** 1 Department of Neurology, Hualien Tzu Chi Hospital, Buddhist Tzu Chi Medical Foundation and Tzu Chi University, Hualien 97002, Taiwan; 2 Department of Biomedical Engineering and Medical Device Innovation Center, National Cheng Kung University, Tainan 70101, Taiwan

**Keywords:** nonlinear analysis, multiscale approximate entropy, gait signals, neurodegenerative diseases

## Abstract

Neurodegenerative diseases such as amyotrophic lateral sclerosis (ALS), Parkinson’s diseases (PD), and Huntington’s disease (HD) are not rare neurological diseases. They affect different neurological systems and present various characteristic gait abnormalities. We retrieved gait signals of the right and left feet from a public domain on the Physionet. There were 13 patients with ALS, 15 patients with PD, 20 patients with HD and 16 healthy controls (HC). We used multiscale approximate entropy (MAE) to analyze ground reaction force on both feet. Our study shows that MAE increases with scales in all tested subjects. The group HD has the highest MAE and group ALS has the lowest MAE. We can differentiate ALS from HC by MAE, while scale factors >10 in the left foot. There are few significant differences of MAE between the HC and HD. We found a good correlation of MAE between both feet in group ALS. In conclusion, our results indicate that MAE analysis of gait signals can be used for diagnosis and long-term assessment for ALS and probably HD. Similarity of MAE between both feet can also be a diagnostic marker for ALS.

## 1. Introduction

Walking steadily involves a complex integration of multiple neurological systems and attributes to proprioception, vestibular system, coordination of agonistic and antagonistic muscles (mainly controlled by the cerebellum and basal ganglia), muscle strength, stability of the skeletal system, neurons for programming and cognition, etc. [1]. Dysfunction of any system results in characteristic patterns of gait abnormality. Therefore, gait evaluation is a very important clinical assessment for patients with neurological diseases [2,3]. Bipedal gait cycle is a useful quantitative analysis of gaits, defined as the time period during locomotion: the time that elapses from one foot contacting the ground until the same foot touching the ground again to propel the person’s center of gravity at the right direction of motion. A single gait cycle, also termed as a stride, is divided into a stance period and swing period depending on the motion of the leg and ground reacting force [4,5]. Several devices are used to record gait signals and many innovative methods have been applied to assess gait cycles [6]. However, multidisciplinary collaborations and funds are necessary to further the development of recording devices and processing systems. Physiobank (http://www.physionet.org/physiobank) is a publicly open database under the auspices of the National Center for Research Resources of the National Institutes of Health [7]. It offers freely available data of many physiological systems, besides the cardiovascular system. It contains well-characterized digitalized biomedical signals, including ECG, blood pressure, gaits, and other biomedical signals from healthy subjects, and patients with neurodegenerative diseases, cardiac arrhythmia, sleep apnea, etc. [7]. Many researchers have used this database to design novel gait analyses for patients with neurodegenerative diseases in an attempt to improve diagnostic accuracy and evaluate progression of diseases [8,9].

It is known that different neurological diseases present characteristic gait patterns based on their various pathophysiological mechanisms. Amyotrophic lateral sclerosis (ALS) affects pyramidal system and results in weakness, but not dyscoordination of movement. Parkinson’s disease (PD) affects the extrapyramidal system and causes slowing of coordination, but no weakness. Huntington’s disease (HD) causes degeneration of the neostriatum. Unlike the clinical features of PD, one of the cardinal presentations of HD is random, jerky, uncontrollable movements termed chorea. These three degenerative diseases present characteristic gait anomalies caused by weakness, bradykinesia, and hyperkinesia. These patients’ gait signals are available in the Physionet databank.

To adapt to the ever-altering environment, several physiological systems have to change frequently to maintain homeostasis. For example, the cardiovascular system continuously regulates the heart rate beat-by-beat to maintain consistent blood pressure via complex integrations of neural circuits and the cardiovascular system [10]. Several methods are used to analyze gait cycle in the time domain to improve diagnostic accuracy and long-term assessments of neurological diseases characterized by gait problems [11]. Approximate entropy is closely related to Kolmogorov entropy, but is simpler and easier to implement. It is a popular method for assessing the regularity of typically short and noisy time series of biological signals [12]. Taking into account multiple scales, multiscale approximate entropy (MAE), defined as a weighted summation of scale-dependent entropy, has the ability to reveal more nuanced changes of complexity [13]. It has been used to assess the degree of discrepancy of acquired signals. For example, a decrease in heart rate entropy was noted in the elderly and patients with systemic illnesses such as diabetes mellitus [13,14,15]. Keeping one’s center of gravity in the midline of a walking path and maintaining a stable gait requires continuous adjustment of the gait, step by step. Studies have used nonlinear analyses to evaluate gait parameters in the time domain, such as stride intervals, or changes of pressure acquired by sensors on the feet [16,17,18]. In this study, we used MAE to quantify the dynamic gait patterns of healthy controls and patients with neurodegenerative diseases.

## 2. Methods

### 2.1. Data Source and Study Protocol

We retrieved gait signals of neurodegenerative diseases from Physionet [7]. The data were acquired by force-sensitive insoles placed in the subjects’ shoes. These sensors recorded changes in force applied to the ground during walking, at a sampling rate of 300 Hz [19]. According to the study protocol, the subjects were instructed to walk at their normal pace along a 77-m hallway for 5 min [20,21]. We selected a 3–4 min segment of stable signals for analysis.

### 2.2. Multiscale Approximate Entropy

Multiscale entropy has been used to analyze the complexity of nonlinear signals in time series. We used MAE to quantify the complexity of pressure signals of both feet retrieved from the Physionet database. Generally nonlinear physiological signals compose a trend [22]. Therefore, non-zero means may be included and cause incorrect analyses. Herein, we used empirical mode decomposition (EMD) to decompose the original signals [23]. Briefly, the steps for the EMD algorithm consists of the following steps: (1) Identifying the extrema (maxima and minima) of the analyzed signals; (2) generating the upper and lower envelopes by an interpolation of the extrema points identified in step 1; (3) making an average computation of the upper and lower envelopes; (4) calculating the difference between the original signals and the means found in step 3 to obtain residual signals, namely intrinsic mode function (IMF); (5) repeating steps 3 and 4 on the IMF till the residual signals become a monotonic function, a residue. The original signals were de-trended by subtracting the residue and last one or two IMFs with very low frequency, empirically. After that, the de-trended data proceeded to MAE analysis. The MAE method is composed of two main procedures, namely coarse-graining [13] and approximate entropy [12].

First, coarse-graining comprises of:

(1) Given the pressure signals on time series as {P1, P2, P3,……P*n*}, consecutive coarse-grained on time series as {*x*(*τ*)} determined by the scale factor *τ*, which was constructed as our previous publication [15]. Briefly, the {*x*(*τ*)} was generated by averaging the data within a non-overlapping window with increasing length, *τ*. In this study, we calculated the complexity of scale factors (*τ* = 1, 2, …, 20) by using approximate entropy.

Second, approximate entropy comprises of:

(2) Define the series {*x*(*τ*)} with length *N* and the two parameters of *m* and *r* (where *m* = Embedded dimension of the vector; *r* = tolerance). Generally, these parameters are used by experience and conditioned by repeated tests. In this study, we used *N* = 3000, *m* = 2 and *r* = 0.2.
(3) Form a sequence vector x(1), x(2), …, x(N−m+1) in $R^{m}$ with *m*-dimensional space by:
  (1) x(i)={u(i+1), u(i+2), u(i+m−1)}, 1≤i≤(N−m+1)
(4) Then we used the sequence x(1), x(2), …, x(N−m+1) to construct:
  (2) $$C_{i}^{m}\;\left(r\right)=\frac{\left(\mathrm{Number}\;\mathrm{of}\;x\left(j\right)\;\mathrm{such}\;\mathrm{that}\;d\left[x\left(i\right),x\left(j\right)\right]\leq r\right)}{\left(N-m+1\right)}$$
(5) In which $d\left[x,x^{*}\right]$ was defined as:
  (3) $$d\left[x,x^{*}\right]=\underset{a}{\max}\left|u\left(a\right)-u^{*}\left(a\right)\right|,$$
(6) Then we define:
  (4) $$\Phi^{m}\left(r\right)=\frac{1}{N-m+1}\;\overset{N-m+1}{\underset{i=1}{\sum }}\;\left(\log C_{i}^{m}\left(r\right)\right)$$
(7) Therefore, approximate entropy (AppEn) can be defined as:
  (5) $$\mathrm{AppEn}=\Phi^{m}\left(r\right)-\Phi^{m}\left(r-1\right)$$

### 2.3. Generation and Analysis of Simulated Gait Signs

Generally, the ground reactions forces were almost similar in each gait cycle. To clearly demonstrate the complexity of gait signals, we generated a series similar to the gait pattern as simulated_gait(t)=asin(2π×f×t+Φ)+bcos(2π×f/2×t)+c, where f=1.2, t=0~10, Φ=π/4, a=0.5, b=2,c=2.5 in this study. In order to compare the MAE between the simulated_gait(t) under the condition of with and without noise, a noise was appended to simulated_gait(t) as simulated_gait_noise(t)=simulated_gait(t)+noise(t), where noise(t) is a randomized signal. The complexity of the gait of a healthy control subject, simulated_gait(t), and simulated_gait(t) were analyzed by MAE as described in Section 2.2.

### 2.4. Statistical Analysis

Data are expressed as mean ± standard deviation (SD). Significant differences in demographic data, gait speed, and MAE between different groups were determined using post hoc Dunnett’s test. The correlation of MAE between the right and left foot were analyzed by Pearson correlation formula. We used SPSS software (Version 14.0, SPSS, Inc., Chicago, IL, USA) for all statistical analyses. A *p* value less than 0.05 was considered statistically significant.

## 3. Results

### 3.1. Age, Sex and Demographic Data of the Subjects

The databank of neurodegenerative diseases recruited 64 subjects, including 16 healthy controls (HC) aged 20–74 (38.69 ± 18.73) years, 13 patients with ALS aged 39–70 (55.62 ± 12.83) years, 15 patients with PD aged 44–80 (67.20 ± 10.69) years, and 20 patients with HD aged 29–71 (47.37 ± 12.51) years. In terms of pathophysiological mechanisms, the ALS group presented gait problems caused by muscle weakness, the HD group had coordination problems associated with hyperkinesia, and the PD group had unsteadiness associated with bradykinesia, which resulted from degeneration of the basal ganglia. Patients with ALS and PD were significantly older than the HC (Table 1). There was female predominance in the groups HC and PD (87.5% and 70.0%, respectively). Patients with ALS had higher body mass indices (BMI) than the HC (25.21 ± 5.35 kg/m^2^ vs. 19.87 ± 2.71 kg/m^2^). The gait speeds were significantly slow in the groups ALS and PD than those in the group HC (1.054 ± 0.218 m/s vs. 1.354 ± 0.160 m/s and 0.999 ± 0.202 m/s vs. 1.354 ± 0.160 m/s).

### 3.2. MAE Analyses of Normal Gait Signals and Simulated Signals

**Figure 1:**
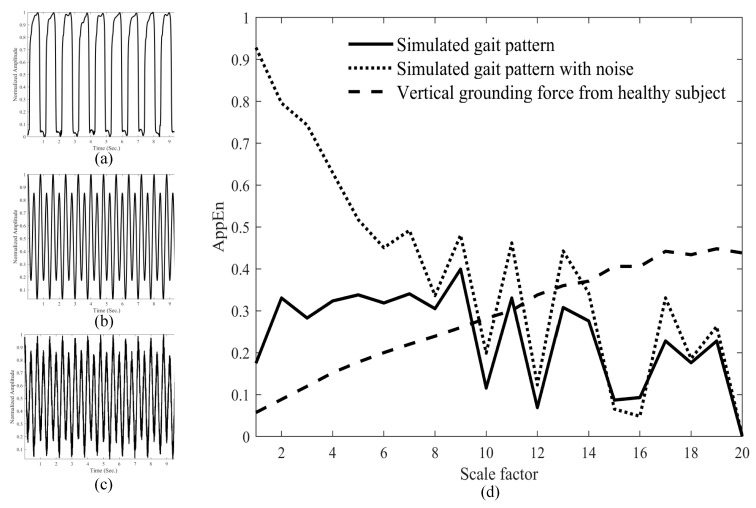
Gait force signals of healthy control 10 (**a**), simulated periodic signals (**b**), simulated periodic signals intermixed with random noises (**c**). Multiscale approximate entropy analysis of gait signals (dashed line), simulated signals (solid line), and simulated signals intermixed with random noises (dotted line). MAE increases with scales in normal gait signals, but is not found in simulated signals (**d**).

Figure 1a shows the gait forces of healthy control 10. The simulated signals and simulated signals, intermixed with random noises, are demonstrated in Figure 1b,c, respectively. Figure 1d shows the MAE analyses of these three signals. Although the gait signals seem regularly periodic, MAE increases with scales in the healthy subject. The MAE does not change obviously in the simulated signals. MAE of the simulated signals intermixed with random noise are higher at small scales, but decreases while the scales increase.

### 3.3. MAE Analysis of Gait Signals in Each Foot

**Figure 2:**
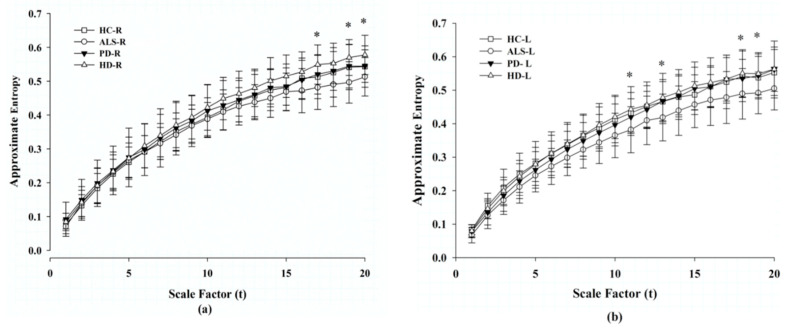
Approximate entropy of the gait signals from scales 1 to 20 among the four groups: (**a**) changes of entropy in the right foot; (**b**) changes of entropy in the left foot. HC: healthy control, ALS: amyotrophic lateral sclerosis, PD: Parkinson’s disease, HD: Huntington’s disease. * *p* < 0.05 between groups HD and ALS. Significant difference determined by post hoc Dunnett’s test.

We used MAE to analyze the gait signals acquired from the patients’ right and left feet. Figure 2 shows the MAE gait analysis for the HC and patients with ALS, PD, and HD. We found that entropy increased with scale factors. Patients with ALS had the lowest MAE and patients with HD had the highest MAE among these four groups. The significant differences in MAE between these two groups only exist at high scales—17, 19, and 20 in the right foot; and 11, 13, 18, and 19 in the left foot. There is no statistical significance in MAE between the healthy controls and patients with ALS, PD, and HD.

### 3.4. Correlation of Approximate Entropy Between the Right and Left Sides.

**Figure 3:**
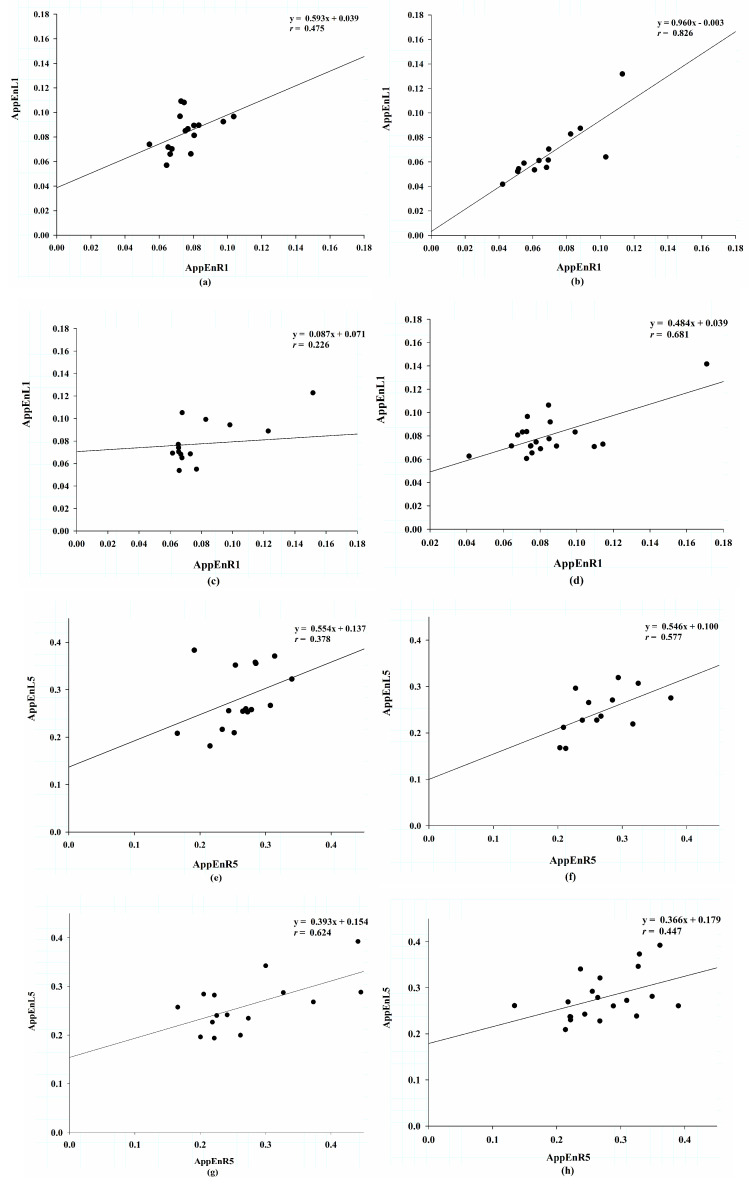
Regression plotting of approximate entropy between the left and right feet at scale factor 1, (**a**) in the healthy controls, (**b**) in the patients with amyotrophic lateral sclerosis, (**c**) in the patients with Parkinson’s disease, (**d**) in the patients with Huntington’s disease; regression plotting of approximate entropy between the left and right feet at scale factor 6, (**e**) in the healthy controls, (**f**) in the patients with amyotrophic lateral sclerosis, (**g**) in the patients with Parkinson’s disease, (**h**) in the patients with Huntington’s disease.

To examine the synergism between feet, which forms the basis of a stable gait, we studied the correlation efficiency of MAE between the right and left feet. Figure 3 shows the correlation between both feet by regression plotting at scale factors 1 and 5. At scale factor 1, the group ALS had the steepest slope (0.906) with a correlation coefficient (*r*) of 0.826; the group HC exhibited a slope of 0.593 and an *r* of 0.475; in the group PD, slope was 0.087 and *r* was 0.226; and in group HD, slope was 0.484, *r* was 0.681 (Figure 3a–d). At scale factor 5, the group ALS still had the steepest slope at 0.546 with an *r* of 0.577; in the group HC, slope was 0.554 and *r* was 0.378 In the group PD, slope was 0.393 and *r* was 0.624; in the group HD, slope was 0.366 and *r* was 0.447 (Figure 3e–h). To quantify the correlation between the right and left gait signals from scale factors 1 to 5, we used the Pearson correlation test (Table 2). The group ALS had the highest correlation coefficients and a decrease with scales (from 0.826 to 0.577). The correlation coefficients did not change with scales in the group HC; there were remarkable increased correlation coefficients in group PD (from 0.226 to 0.624); in contrast, the correlation coefficients decreased with scales in group HD (from 0.681 to 0.447). We also used the slopes of linear regression to represent the similarity of MAE between the right and left feet. The group ALS had the steepest slopes decreased with scales (from 0.906 to 0.546). The shallower slopes existed in groups PD and HD. The group PD had the shallowest slope at scale 1 (0.087), then unchanged from scales 2 to 5 (from 0.303 to 0.393). On the other hand, slope was 0.484 at scale 1 and did not change with scales (from 0.345 to 0.366) in group HD.

## 4. Discussion

Gait dysfunction is a common problem in the elderly and patients with neurodegenerative diseases. In addition to acquiring biophysical signals via pressure sensors, accelerators or video, gait analysis with mathematical algorithms is another important future application [24]. Physionet offers a useful and easy-to-access databank that researchers can access to develop and evaluate validations of innovative analytic methods [25]. However, there are some limitations. First, because of small sample size, heterogeneous clinical manifestation, and staging, it is not easy to obtain good clinical correlation with the analyzed results. It is not possible to show a correlation between the severities of disease and the analytical results. Most research articles have demonstrated the difference between the disease groups and the healthy controls [26,27]. Only one paper gave details of clinical manifestations. The author did not show any correlation between the analyzed results and clinical severities [8]. Second, the number of tested subjects in each group is not sufficient to attain a statistical power. Although there is no statistical significance to the findings, trends in the analytical changes might indicate useful methods for future clinical applications.

Bradykinesia is a cardinal clinical feature of PD. It is also found in patients with HD and ALS [28,29]. The data in Table 1 reveal that gait speeds were significantly slower in patients with ALS and PD and tended to decrease in the group HD. The sample size was small, but this result indicated that the gait data of the recruited subjects in this study might be similar to those of the population-based survey. However, it should be taken into account that subjects in the group HC were significantly younger than patients with ALS and group HD. Meanwhile, the group HC contained 87.5% female subjects, and 70.0% of the recruited PD patients were female. Age and sex may be two confounding factors influencing the results of gait analysis and complexity [30,31,32].

It has been found that multiscale entropy of stride intervals decreased with scales in paced walking in healthy volunteers [17]. Lipsitz and colleagues proposed the “Loss of Complexity” theory, which hypothesized that aging, degeneration, or diseases were associated with decreases in the complexity of the signals of different physiological systems, such as heart rate variability and fluctuation in blood pressure, etc. [33,34]. This phenomenon has been found in many diseases. In contrast to entropy of stride intervals [17], Figure 2 shows that the MAE of our analyzed pressure signals increased with scale factors in the groups HC, ALS, PD, and HD. These were acquired by insoles placed in the subject’s shoes at a sampling rate of 300 Hz. After the coarse-graining procedure, various consecutive force signals within a non-overlapping window (*τ*) were averaged to form a new series. Approximate entropy was then applied to these new series. Unlike entropy analysis of stride time or texture classification, the MAE of ground reaction force in this study quantified the complexity of force signals at various time windows. Because of an inertial effect, the swing of the gravity center increases. Therefore, both feet change forces to dynamically maintain the gravity center at the midline. Adaptability increases with time; complexity increases with scales. The MAE results demonstrate subtle changes in the complexity of gait forces. To make a point, we generated signals similar to the left-foot signals of control subject 10 and added white noises to increase complexity of the simulated signals (Figure 1a–c). We found that even on adding white noises to increase complexity, the MAE of the simulated signals did not increase with scales (Figure 1d).

Patients with ALS had the lowest MAE. That might be due to progressive weakness of the limbs, which made these patients lose the ability to adjust miniature movements and maintain stability during changes in weight bearing on their right and left feet. On the other hand, HD causes hyperkinetic movement of the feet, and therefore, resulted in the highest MAE among the four studied groups. Increased complexity of stride intervals in patients with ALS was noted in previous publications by using Poincaré plotting [35] or fluctuation magnitude [21]. Our results show that the differences between these two groups increase with scales, although there was no statistical significance. As mentioned previously, it might be due to the small sample size. However, the contrast between our findings and the published results could be that weakness of ALS loses the ability to adjust step-by-step variation of the gravity center, resulting in attenuated homeostasis of the ground reaction force. On the other hand, loss of adaptability of gait force results in a decrease in the ability to maintain center of gravity at the right position and an increase in the complexity of stride interval to prevent subsequent falling. MAE of ground reaction force might be less sensitive to differentiation of ALS than stride intervals. However, large and well-designed studies with relevant clinical features are necessitated. The notable changes of MAE of ground reaction force in ALS and HD at large scales may be related to pathophysiological mechanisms and can be used as an indicator for differential diagnosis and long-term assessment of these two diseases. There is no significant difference in MAE between the groups HC and PD. Because PD patients are not characterized by motor weakness, we propose that patients with PD, even those with bradykinesia, retain the ability to maintain gait stability in most cases. Afsar and colleagues used three different complexity measurements—Shannon, Kulback-Leibler and Limontovich’s renormalization entropies—to analyze ground reaction forces of the same databank. They found that only the last measurement had high sensitivity to differentiate PD from HC and good correlation with clinical severity [36]. Our results show the potential uses of MAE for gait analysis. This method may be applied in early diagnosis and long-term follow-up for patients with ALS and HD, but not PD.

To look into the correlation of entropies between the right and left feet, Figure 3 shows regression plotting of MAE at scale factors 1 and 5 in these four groups. It discloses an interesting finding that there was a remarkable good relationship between both feet in group ALS. The group HD had the lowest correlation efficient at scale 1, but the slopes increased at scale 5, and the correlation became significant. The changes of correlation became clearer when we used Pearson correlation analysis scale by scale. Table 2 lists the correlation coefficients of approximate entropy between both feet for scale factors ranging from 1–5 in each group. The group ALS had the highest correlation coefficient from scales 1–5 among the four groups. The correlation coefficients decreased with scales in groups ALS and HD, and increased with scales in the group PD. Linkages between these trends and pathophysiological mechanisms remain uncertain. The slopes of approximate entropy between the right and left feet from scales 1–5 in each group are also demonstrated in Table 2. The slopes may represent the similarity of entropy between both feet. The group ALS had the steepest slopes, which decreased with scales. As proposed previously, patients with ALS have lost the complexity of gait pressure signals between both feet. Even so, they tend to adapt to overcome this problem, resulting in minor increase in complexity of ground reaction force in each foot. In contrast, patients with HD had the shallowest slopes and increased with scales. This finding may suggest that HD patients have a tendency to approach higher similarity of entropy in both feet. There were no significant differences in approximate entropy between the right and left feet at scales 1 and 2 in group PD. The correlation coefficients became significant and the slopes increased with scales at scales 3 to 5. These changes may suggest that although the patients with PD did not show weakness, they lose complexity on long walks. This may be relevant to the characteristic festinating gait in patients with PD. Because of bradykinesia, patients cannot maintain a gravity center at the midline, when walking long distances. Large-scale clinical studies with good stratification of severity may prove our hypothesis. Our findings suggest that similarity of ground reaction force between both feet may be helpful for early diagnosis, long-term follow-up, and assessments of therapeutic efficacy for patients with ALS, PD, and HD.

## 5. Conclusions

The results of our study demonstrate the usefulness of MAE in gait analysis for neurodegenerative diseases. Changes in MAE reveal a clinically relevant phenomenon among healthy subjects, and patients with neurodegenerative diseases characterized by bradykinesia, weakness, and hyperkinetic movements. We also found a similarity in MAE between the right and left feet, which is a characteristic feature of ALS, PD, and HD.

## Figures and Tables

**Table 1:** Demographic data of the subjects.

	HC (*n* = 16)	ALS (*n* = 13)	PD (*n* = 15)	HD (*n* = 20)
Age (years)	38.69 ± 18.73	55.62 ± 12.83 *	67.20 ± 10.69 **	47.37 ± 12.51
Sex (Male %)	12.5%	66.7%	30.0%	76.9%
Height (m)	1.833 ± 0.087	1.7446 ± 0.950	1.87 ± 0.152	1.8437 ± 0.089
Weight (kg)	66.81 ± 11.08	77.12 ± 21.15	75.07 ± 16.90	73.47 ± 16.24
BMI (kg/m^2^)	19.87 ± 2.71	25.21 ± 5.35 *	21.21 ± 2.64	21.55 ± 4.44
Gait speed (m/s)	1.354 ± 0.160	1.054 ± 0.218 *	0.999 ± 0.202 **	1.15 ± 0.349

Data are expressed as mean ± SD. HC: healthy control, ALS: amyotrophic lateral sclerosis, PD: Parkinson’s disease, HD: Huntington’s disease, BMI: body mass index. * *p* < 0.05 between HC and ALS, ** *p* < 0.05 between HC and PD. Significance of difference determined by post hoc Dunnett’s test.

**Table 2:** Correlations coefficients and regression slopes of approximate entropy between the right and left feet from scale factors 1 to 5 in each group.

	HC			ALS			PD			HD		
	*r*	Slope	*p*	*r*	Slope	*p*	*r*	Slope	*p*	*r*	Slope	*p*
AppEnR1/L1	0.475	0.593	0.063	0.826	0.906	0.001 *	0.226	0.087	0.417	0.681	0.484	0.001 *
AppEnR2/L2	0.392	0.458	0.133	0.793	0.812	0.001 *	0.494	0.303	0.061	0.536	0.345	0.015 *
AppEnR3/L3	0.372	0.466	0.156	0.694	0.576	0.008 *	0.53	0.363	0.042 *	0.494	0.379	0.027 *
AppEnR4/L4	0.397	0.58	0.128	0.656	0.616	0.015 *	0.544	0.395	0.036 *	0.42	0.351	0.065
AppEnR5/L5	0.378	0.554	0.149	0.577	0.546	0.039 *	0.624	0.393	0.013 *	0.447	0.366	0.048 *

HC: healthy control, ALS: amyotrophic lateral sclerosis, PD: Parkinson’s disease, HD: Huntington’s disease, AppEnR1/L1: the relationship between approximate entropy of gait signals in the right and left feet at scale 1, AppEnR2/L2: the relationship between approximate entropy of gait signals in the right and left feet at scale 2, AppEnR3/L3: the relationship between approximate entropy of gait signals in the right and left feet at scale 3, AppEnR4/L4: the relationship between approximate entropy of gait signals in the right and left feet at scale 4, AppEnR5/L5: the relationship between approximate entropy of gait signals in the right and left feet at scale 5, *r*: the correlation coefficient calculated by Pearson coefficient formula, slope: the regression slope estimated by linear regression. * *p* < 0.05 by Pearson correlation.
